# *Corvisyringophilus*, a New Genus in the Family Syringophilidae (Acariformes: Prostigmata) and Its Phylogenetic Position among Primitive Genera †

**DOI:** 10.3390/ani14192790

**Published:** 2024-09-26

**Authors:** Maciej Skoracki, Ólafur K. Nielsen, Bozena Sikora

**Affiliations:** 1 Department of Animal Morphology, Faculty of Biology, Adam Mickiewicz University, Uniwersytetu Poznańskiego 6, 61-614 Poznań, Poland; 2 Icelandic Institute of Natural History, Urriðaholtsstræti 6-8, IS-210 Garðabær, Iceland; olafur.k.nielsen@natt.is

**Keywords:** Acari, birds, ectoparasites, *Corvus*, quill mites, raven

## Abstract

**Simple Summary:**

The Syringophilidae family is one of the most diverse groups of prostigmatic mites (Acari: Prostigmata) parasitising birds. In this paper, we describe a new genus and species, *Corvisyringophilus krummi*, which parasitises the Common Raven (*Corvus corax*) in Iceland. This new genus belongs to a group of primitive genera that retain a complete set of idiosomal and leg setae. Our phylogenetic analysis suggests that *Corvisyringophilus* is morphologically closely related to the genera *Blaszakia* Skoracki & Sikora, 2008 and *Charadriphilus* Bochkov & Mironov, 1998, which are associated with birds of different and phylogenetically unrelated orders: Musophagiformes and Charadriiformes, respectively. This research highlights the importance of examining the genus *Corvus* in order to better understand the specialised host–parasite relationships. Furthermore, the study emphasises the significance of using museum collections to study parasitic interactions.

**Abstract:**

Syringophilidae is one of the most species-rich families in the superfamily Cheyletoidea, comprising approximately 420 species across 62 genera and two subfamilies. In this paper, we propose a new genus, *Corvisyringophilus*, and a new species, *C. krummi* gen. n. et sp. n., found in the wing covert quills of the Common Raven, *Corvus corax* Linnaeus, in Iceland. *Corvisyringophilus* is placed among the primitive genera of syringophilid mites, which possess the full complement of idiosomal and leg setae. Phylogenetic analysis based on morphological characters suggests that this genus forms a sister clade to *Blaszakia* Skoracki & Sikora, 2008, and *Charadriphilus* Bochkov & Mironov, 1998, which inhabit birds of the orders Musophagiformes and Charadriiformes, respectively. The study proposes that the current distribution patterns of quill mites, based on their morphological characteristics, may result from multiple host switching followed by co-speciation events, highlighting the complex evolutionary dynamics within this family.

## 1. Introduction

The Syringophilidae is one of the most species-rich families in the superfamily Cheyletoidea (Acariformes: Prostigmata), and currently comprises approximately 420 species arranged in 62 genera and two subfamilies, Picobiinae Johnston & Kethley, 1973 and Syringophilinae Lavoipierre, 1953 [1,2,3,4,5,6]. Syringophilid species have been recorded from 25 neognathous bird orders (Neognathae) and 1 paleognathous order, Tinamiformes (Paleognathae) [7]. These obligatory ectoparasites inhabit quill feathers and exhibit high specificity to both their hosts and habitats. Consequently, individual species infest a limited range of hosts and specific types of feathers [3,4,8].

Among quill mite genera, there are highly speciose taxa, such as *Picobia* Haller, 1878, which comprises 44 species; *Syringophiloidus* Kethley, 1970, with 47 species; and *Syringophilopsis* Kethley, 1970, with 49 species. There are also monotypic genera, such as *Trypetoptila* Kethley, 1970, *Tanopicobia* Skoracki et al., 2020, and *Calamincola* Casto, 1978. Most genera are associated with specific avian orders; for example, *Gunabopicobia* Skoracki & Hromada, 2013, is associated exclusively with Columbiformes; *Aulobia* Kethley, 1970, is associated with Passeriformes; and *Bubophilus* Philips & Norton, 1978, is associated with Strigiformes [3,4]. However, some genera are distributed across a broad range of host orders, which are not always phylogenetically closely related. For instance, *Peristerophila* Kethley, 1970, is associated with Accipitriformes, Bucerotiformes, Columbiformes, Coraciiformes, Falconiformes, and Psittaciformes; *Picobia* is recorded on Bucerotiformes, Passeriformes, and Piciformes; and *Rafapicobia* Skoracki, 2011, is noted on Coraciiformes, Gruiformes, Passeriformes, Piciformes, and Psittaciformes [3,4,5].

Compared to Cheyletidae Leach, 1815, their sister family, the Syringophilidae, has relatively uniform morphology. The main criterion for distinguishing the genera is the presence or absence of setae on the legs and idiosoma [9,10,11]. However, seta characters are crucial for the phylogenetic reconstruction of the whole Eleutherengones group [12]. Among syringophilids, some genera retain a full complement of setae, while others lose certain setae [3]. The absence of idiosomal setae is notable on the propodonotum, such as setae *vi* in genera like *Aulonastus* Kethley, 1970, *Neoaulonastus* Skoracki, 2004, and *Peristerophila*; or on the opisthosoma, such as aggenital setae *ag2* in *Kethleyana* Kivganov, 1995, *Mironovia* Chirov & Kravtsova, 1995, and *Picisyringophilus* Skoracki & OConnor, 2010; pseudanal setae *ps2* in *Betasyringophiloidus* Skoracki, 2011, or *Krantziaulonastus* Skoracki, 2011, and genital setae *g2* in *Procellariisyringophilus* Schmidt & Skoracki, 2007. The absence of leg setae is more varied and can include the loss of setae on different podomers and pairs of legs, such as the absence of tarsus setae *vsI* in *Paraniglarobia* Skoracki, 2011, or *Philoxanthornea* Kethley, 1970, *vsII* in *Cuculisyringophilus* Skoracki, 2008, or *Niglarobia* Kethley, 1970; femoral setae *dFII* in *Fritschisyringophilus* Bochkov et al., 2004, or *Meitingsunes* Skoracki & Glowska, 2010, *dFIII–IV* in *Peristerophila*, or *Psittaciphilus* Fain et al., 2000; genu setae *dGII* in *Syringophiloidus*, or *Galliphilopsis* Skoracki & Sikora, 2004, *l’GIV* in *Neosyringophilopsis* Skoracki & Sikora, 2005, or *Apodisyringiana* Skoracki, 2005; tibial setae *dTIII–IV* in *Galliphilopsis*, or *Neoaulonastus*, and trochanter setae *l’RI–II* in *Aulonastus*, or *Picobia*. In syringophilid genera, species are primarily distinguished by quantitative characteristics such as the length of the stylophore or setae, with a few exceptions, and this is the main reason why the genera are not divided into subgenera.

In the present paper, we propose a new genus, *Corvisyringophilus,* for a new species, *C. krummi* gen. n. et sp. n., collected from the feather quills of the Common Raven *Corvus corax* Linnaeus in Iceland, and determine the placement of this new genus among other primitive genera of syringophilid mites, which retain a full complement of setae. It should also be noted that this is the fourth species of quill mite recorded on bird hosts in Iceland, the other three being *Mironovia lagopus* Bochkov & Skirnisson, 2011, from the Rock Ptarmigan *Lagopus muta* (Montin) (Galliformes: Phasianidae); *Syringophilus bipectinatus* Heller, 1880, from the Red Junglefowl (domestic type) *Gallus gallus domesticus* (Linnaeus); and *Stibarokris nielseni* Skoracki et al., 2022, from the Manx Shearwater *Puffinus puffinus* (Brünnich) [13,14,15,16].

## 2. Materials and Methods

### 2.1. Mites Collection and Description

Mite material was collected from the dry skin of the Common Raven *Corvus corax* housed in the ornithological collection at the Icelandic Institute of Natural History, Reykjavík, Iceland (IINH). Mites were removed from the quills of small wing coverts using sharp tweezers. Initially, mite specimens were softened and cleared in Nesbitt’s solution at 40 °C for about two days and then mounted in Hoyer’s medium [17]. Identification of mite specimens and drawing preparations were carried out with a ZEISS Axioscope2™ light microscope, equipped with differential interference contrast (DIC) optics and a camera lucida (Carl-Zeiss AG, Oberkochen, Germany). In the description, all measurements are given in micrometres, with ranges for paratypes in parentheses following data for a holotype. The general syringophilid morphological terms follow Kethley [2] and Skoracki [4]. The nomenclature for leg setation is that of Grandjean [18]; the idiosomal setation follows Grandjean [19], as adapted for Prostigmata by Kethley [20].

Specimen depositories and reference numbers are cited using the following abbreviations: AMU—Adam Mickiewicz University, Department of Animal Morphology, Poznan, Poland; IINH—Icelandic Institute of Natural History, Reykjavík, Iceland.

### 2.2. Phylogenetic Analysis

To determine the placement of the new genus, we used the data matrix recently published by Skoracki et al. [7]. The species used in the analyses represent the most primitive genera of the family Syringophilidae, specifically the 19 genera that possess a full complement of setae on the idiosoma and legs. Because the monophyly of the family Syringophilidae has been tested with numerous outgroups and always received high support [9,10,21,22], only two outgroups were used in the analyses, both belonging to the sister family Cheyletidae—the quill-inhabiting predator *Cheletopsis norneri* (Poppe) and the free-living predator *Cheyletus eruditus* (Schrank). In the cladistic analysis, only qualitative characteristics from external morphology were used, focusing on features such as the presence or absence of specific structures and the form of particular morphological features. A total of 32 operational taxonomic units (OTUs) and 50 informative characters were included in the maximum parsimony-based cladistic analysis (Supplementary Tables S1 and S2). All characters in the data matrix were treated as unordered, with their states polarised through outgroup comparison. Phylogenetic relationships were reconstructed using PAUP 4.0 [23], employing a heuristic search for the maximum parsimony analysis. The delayed transformation (DELTRAN) option was applied to favour parallelism over reversal, optimising character states and tracing changes across lineages a posteriori. All characters were analysed without weighting.

## 3. Results

### 3.1. Systematic

Family: Syringophilidae Lavoipierre, 1953.

Subfamily: Syringophilinae Lavoipierre, 1953.

#### 3.1.1. Description

##### Genus *Corvisyringophilus* gen. n

Diagnosis. Female. Small-sized syringophilids. Gnathosoma. The hypostomal apex is rounded and without protuberances. Two pairs of large hypostomal lips are present. Lateral hypostomal teeth are absent. The peritremes are M-shaped with clearly visible chambers in each branch. The anterior tip of each movable cheliceral digit is edentate. The stylophore is rounded posteriorly. Idiosoma. The propodonotum bears six pairs of propodonotal setae arranged in the pattern 2–1–1–2; bases of setae *se* are situated on the propodonotal shield and posterior to *c1*. Setae *d1* are situated closer to *d2* than to *e2*. The hysteronotal shield is present. The aggenital setal series consists of three pairs of setae. Each pseudanal and genital series has two pairs of setae. All idiosomal setae are smooth and whip-like. Legs. Legs I are thicker than II. Antaxial and paraxial members of the claw pair are subequal in size and shape and without a basal angle. Apodemes I are parallel and not fused to apodemes II. Legs contain the full complement of setae.

Male. Features the same as in the female, except hypostomal lips, which are small-sized; stylophore is constricted posteriorly; six pairs of propodonotal setae are arranged in the pattern 2–1–1–1–1; bases of setae *se* are situated out of the propodonotal shield and anterior to *c1*; apodemes I are strongly divergent and not fused to apodemes II.

##### Habitat

Quills of secondary wing coverts.

##### Type Species

*Corvisyringophilus krummi* sp. n.

##### Remarks

Phylogenetic and morphological analyses indicate that *Corvisyringophilus* gen. n. is the most similar to two genera, i.e., *Blaszakia* Skoracki & Sikora, 2008, associated with birds of the family Musophagidae (Musophagiformesthe) [24] and *Charadriphilus* Bochkov & Chistyakov, 2001 associated with birds of the orders Charadriiformes and Gruiformes [25]. In females of these three genera, the lateral hypostomal teeth are absent; the distal tip of the movable cheliceral digits are edentate; the peritremes are M-shaped; bases of setae *se* are situated on the propodonotal shield and posterior to setal bases *c1*; the hysteronotal shield is present and fused to the pygidial shield, its anterior margin reach bases of setae *d2*; the aggenital series contains three pairs of setae; each pseudanal and genital series contains two pairs of setae, each; apodemes I and II are not fused; the idiosoma and legs contain the full complement of setae. The differences between *Corvisyringophilus* and those mentioned above, which are closely related genera, are presented in Table 1.

##### Etymology

The name “*Corvisyringophilus*” is derived from the generic name of the host—*Corvus*—and the type genus of the syringophilid family—*Syringophilus*.

##### *Corvisyringophilus krummi* sp. n. (Figure 1 and Figure 2)

**Figure 1:**
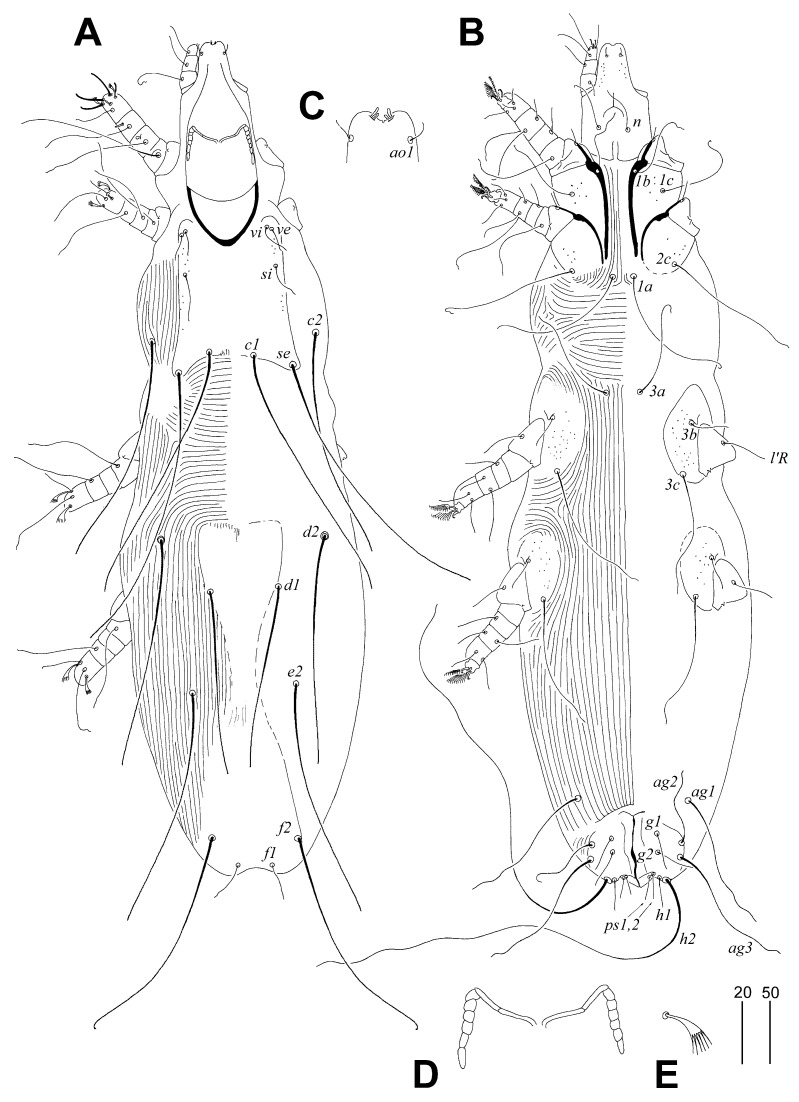
*Corvisyringophilus krummi* gen. n. et sp. n. Female. (**A**) Dorsal view; (**B**) ventral view; (**C**) hypostomal apex in dorsal view; (**D**) peritremes; (**E**) fan-like seta *p’III*. Scale bars (**A**,**B**) = 50 µm, (**C**–**E**) = 20 µm.

Description. Female, holotype. The total body length of the holotype is 655 (650–665 in seven paratypes). Gnathosoma. The infracapitulum is punctate in the anterior part. The hypostomal apex is without protuberances, but two pairs of finger-like hypostomal lips are present. The stylophore is rounded posteriorly and 165 (150–170) long. The exposed portion of the stylophore is apunctate and 125 (115–135) long. Each branch of the peritremes has two chambers, and each lateral branch has six chambers. Idiosoma. The propodonotal shield is entire, with concave anterior and posterior margins, punctate near lateral margins, bearing bases of all propodonotal setae except *c2*. Setae *vi*, *ve*, and *si* are short; the length ratio of setae *vi*:*ve*:*si* is 1:1–1.3:1–2. The bases of setae *c1* are situated anterior to the level of setal bases *se*. Setae *c1* and *se* are subequal in length. The apunctate hysteronotal shield is not fused to the pygidial shield; the anterior margin reaches the level of setae *d2*, the posterior margin tapers, and the bases of setae *d1* are situated on the lateral margins of this shield. The length ratio of setae *d2*:*d1*:*e2* is 1.2–1.3:1:1–1.2. The pygidial shield is apunctate and with an indiscernible anterior margin. Bases of setae *f2* are situated distinctly anterior to the level of setal bases *f1*. Setae *f2* are about six times longer than *f1*; setae *h2* are about twice as long as *f2*. Setae *ag1* and *ag3* are subequal in length, and both pairs of these setae are about twice as long as *ag2*. The genital plate is present, bearing setae ag2 and ag3 bases on its lateral margins. Both pairs of pseudanal setae *ps1* and *ps2* are subequal in length. Both pairs of genital setae *g1* and *g2* are subequal in length, and both pairs are 1.7–2 times longer than pseudanal setae. Coxal fields I and II are sparsely punctate; III and IV are densely punctate. The cuticular striations of the idiosoma are as shown in Figure 1A,B. Legs. Fan-like setae *p*′ and *p*″ of tarsi III and IV have six to eight tines. Setae *tc″III–IV* are about twice as long as *tc’III–IV*. Setae *l’RIII* and *l’RIV* are subequal in length. The lengths of the setae are as follows: *vi* 20 (20–30), *ve* 20 (20–25), *si* 30 (30–40), *se* 185 (180–200), *c1* 205 (175–195), *c2* 185 (180–190), *d1* 145 (140–155), *d2* 180 (180–185), *e2* 180 (160–180), *f1* 30 (25–30), *f2* 185 (175–185), *h1* 30 (25–30), *h2* (350–370), *ag1* 120 (110–125), *ag2* 60 (60–70), *ag3* 125 (125–130), *ps1* and *ps2* 15 (15–20), *g1* and *g2* 30 (25–30), *tc’III–IV* 30 (30–35), *tc″III–IV* 60 (55–60), *l’RIII–IV* 30 (30–35).

**Figure 2:**
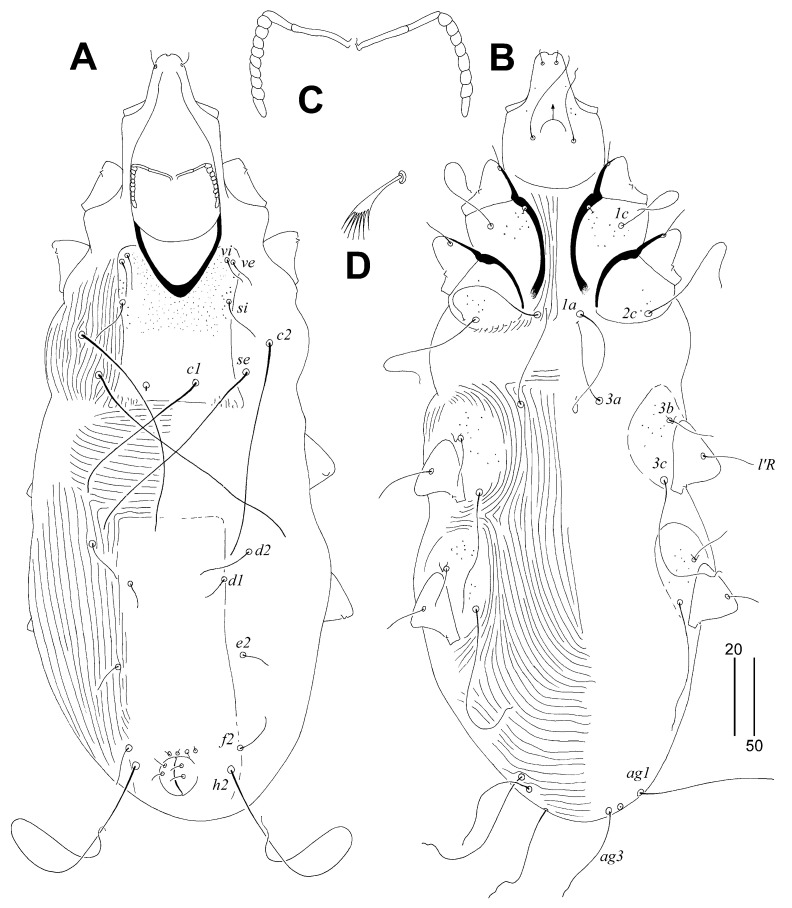
*Corvisyringophilus krummi* gen. n. et sp. n. Male. (**A**) Dorsal view; (**B**) ventral view; (**C**) peritremes; (**D**) fan-like seta *p’III*. Scale bars (**A**,**B**) = 50 µm, (**C**,**D**) = 20 µm.

Male. The total body length is 480–490 in four paratypes. Gnathosoma. The infracapitulum is sparsely punctate in the anterior part. The hypostomal apex is without protuberances. The stylophore is slightly constricted posteriorly and is 150–155 long. The exposed portion of the stylophore is apunctate and 120–125. Each branch of the peritremes has two chambers, each lateral branch has eight or nine chambers. Idiosoma. The propodonotal shield is entire, with a concave anterior margin, covered with minute punctuations in the anterior part, bearing bases of setae *vi*, *ve*, *si*, and *c1*. Setae *vi*, *ve*, and *si* are short and subequal in length. The bases of setae *c1* are situated slightly posterior to the level of setal bases *se*. The hysteronotal shield is fused to the pygidial shield, apunctate; the anterior margin reaches above the level of setae *d2*, and bases of setae *d1*, *f2*, and *h2* are situated on this shield. Setae *d2* are about twice as long as *d1* and *e2*. Setae *h2* are about four times longer than *f2*. All coxal fields are sparsely punctate. The cuticular striations of the idiosoma are as shown in Figure 2A,B. Legs. Fan-like setae *p*′ and *p*″ of tarsi III and IV have six or seven tines. Setae *tc*″*III–IV* are about twice as long as *tc’III–IV*. The lengths of the setae are as follows: *vi* 20–25, *ve* 20–30, *si* 20–30, *se* 130–140, *c1* is variable in lengths: 65 or 100–130, *c2* 110–145, *d1* 15–20, *d2* 30–40, *e2* 15–20, *f2* 25–35, *h2* 230, agenital setae are variable in lengths: *ag1* 30–90, *ag2* 40–70, *ag3* 50–90, *tc’III–IV* 20–25, *tc″III–IV* 40, *l’RIII* 40, *l’RIV* 25, *3b* and *4b* 25, *3c* and *4c* 75.

##### Type Material

Female holotype and paratypes: seven females and four males collected from the quill of the wing covert of the Common Raven *Corvus corax* Linnaeus (Passeriformes: Corvidae) (host reg. no. IINH-RM 10049, male); Iceland: Borgarhofn, South District, East Skaftafell County (Borgarhöfn, Suðursveit, A.-Skaft.) 64°11′32.1″ N 15°48′33.1″ W, 5 October 1989, bird coll. S. Valtýsson, mites removed by M. Skoracki.

##### Type Material Deposition

The holotype and most paratypes (reg. no. MS 24-0903-001) are deposited in the AMU except three female paratypes and one male paratype in the IINH.

##### Etymology

The species name “*krummi*” is derived from the Icelandic word “krummi,” the nickname of the “raven” that is affectionally used by Icelanders when referring to this bird species. This name was chosen to honour the Common Raven, *Corvus corax*, the bird species with which this mite is associated. The use of “krummi” reflects both the common name of the bird in Icelandic culture and the genus *Corvus*, which signifies the host genus of the new species.

#### 3.1.2. Parsimony Analysis

**Figure 3:**
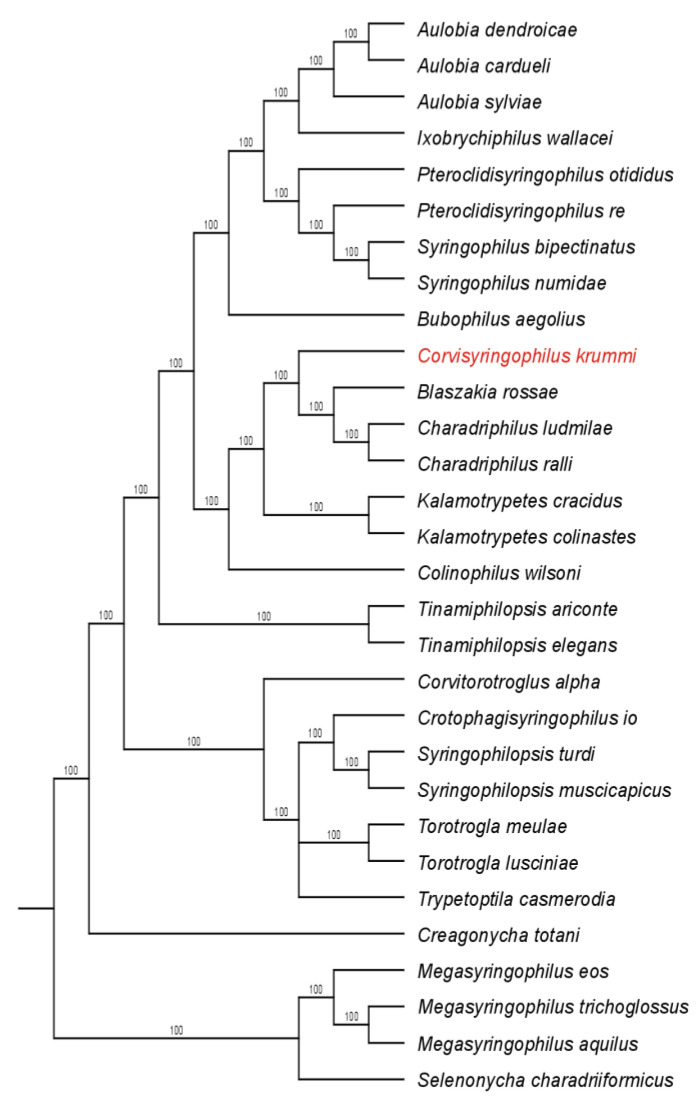
Majority rule consensus of the three most parsimonious trees (tree length 129, CI for only informative characters—0.45, RI—0.69, RC—0.31.) found using the heuristic search option for the unordered and unweighted dataset. Numbers above branches = % trees with the respective node.

**Figure 4:**
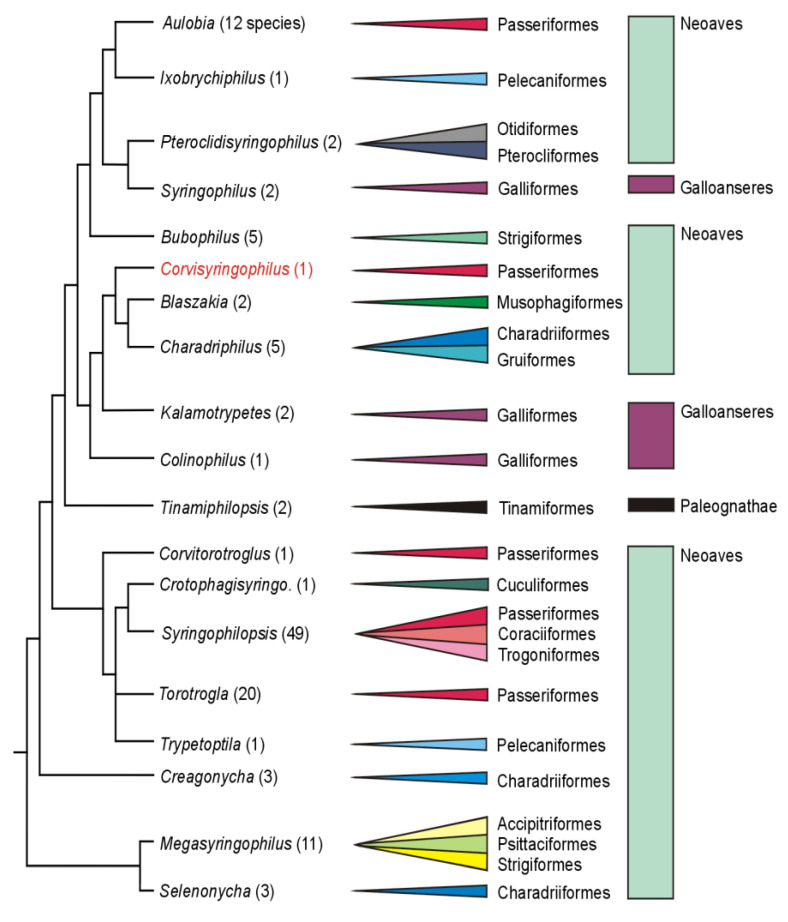
The most parsimonious tree of the primitive syringophilid genera (genera with the full complement of setae) and their host associations (orders of birds). The numbers at the genera names indicate the species in each genus.

Three equally parsimonious trees were produced by the initial analysis, with all characters treated as unordered and unweighted (tree length 129, consistency index (CI) for phylogenetically informative characters—0.45, retention index (RI)—0.69, rescaled consistency index (RC)—0.31. The strict consensus of these trees is shown in Figure 3 and Figure 4. The differences between these trees lie solely in the positioning of the genus *Trypetoptila* relative to the genera *Torotrogla*, *Syringophilopsis*, and *Crotophagisyringophilus*.

## 4. Discussion

Crown birds (Neornithes), comprising approximately 11,000 species, are divided into two distinct monophyletic groups: Palaeognathae, which includes tinamous and ratites, and Neognathae, which encompasses all other avian species [26,27]. Within the Neognathae, the Galloanserae (which consists of Galliformes and Anseriformes) is identified as the sister group to the remaining birds, collectively referred to as Neoaves [28,29,30,31]. Syringophilid mites, from the subfamilies Syringophilinae and Picobiinae, have been found in 27 out of the 44 recognised orders of extant neognathous and palaeognathous birds [5,7].

Syringophilid mites from the most primitive genera, which possess a complete set of idiosomal and leg setae, are distributed across all three major clades of birds: Palaeognathae, Galloanserae, and Neoaves. The presence of syringophilids on these two primary bird clades led Skoracki et al. [32] to suggest that syringophilids likely formed relationships with the ancestors of birds, feathered theropod dinosaurs like *Archaeopteryx* or *Aurornis* [33,34,35,36]. However, recent cladistic analyses indicate that mite genera associated with the earliest divergent clades of extant birds, Tinamiformes (Palaeognathae) and Galloanserae (Anseriformes and Galliformes), are mosaically distributed within the core of the tree (see Figure 4). This contradiction between the presumed syringophilid parasitism of the common bird ancestor and the observed phylogenetic pattern could be explained by multiple host switching (secondary infestation) from Neoaves to palaeognathous and galloanserae birds, followed by subsequent co-speciation [7]. The genus *Corvisyringophilus*, proposed herein, belongs to a clade that includes the genera *Kalamotrypetes* Casto, 1980 and *Colinophilus* Kethley, 1973, found on birds of the order Galliformes [37,38]. It represents a sister clade to the closely related genera *Blaszakia*, which inhabits birds of the order Musophagiformes [24], and *Charadriphilus* found on birds of the orders Charadriiformes and Gruiformes [25,39,40,41] (Figure 3 and Figure 4). Phylogenetic analyses of birds do not indicate a close relationship between Passeriformes and either Musophagiformes or Charadriiformes and Gruiformes [30,31,42]. In this case, we are likely observing the host switching between birds belonging to unrelated orders, followed by subsequent speciation.

The genus *Corvisyringophilus* is one of the several syringophilid genera inhabiting passeriform birds. The order Passeriformes represents the largest and most diverse group of birds, comprising approximately 6,200 species, which constitutes about 60% of all extant avian species. [43]. Notably, this order also harbours the greatest number of syringophilid mite species. To date, 17 genera (out of 65, or 26%) and 241 species (out of 425, or 57%) of syringophilid mites have been documented from passerines belonging to 62 families (out of 143, or 43%). Among the genera parasitising passerine birds, several are exclusively associated with this order, including *Aulobia* Kethley, 1970, *Aulonastus* Kethley, 1970, *Betasyringophiloidus* Skoracki, 2011, *Corvisyringophilus* gen. n., *Fritschisyringophilus* Bochkov, Fain & Skoracki, 2004, *Torotrogla* Kethley, 1970, and *Phipicobia* Glowska & Schmidt, 2014 [2,3,40,44]. In the remaining genera, the majority of species are primarily associated with passerines, with only a small fraction exhibiting host switching to non-passeriform birds: *Krantziaulonastus* Skoracki, 2011, *Neoaulonastus* Skoracki, 2004, *Neosyringophilopsis* Skoracki & Sikora, 2005, *Syringophiloidus* Kethley, 1970, *Syringophilopsis* Kethley, 1970, *Neopicobia* Skoracki, 2011, *Picobia* Haller, 1878, *Pipicobia* Glowska & Schmidt, 2014, and *Rafapicobia* Skoracki, 2011 [2,3,4,45,46]. Interestingly, in two instances, passerines of the family Paradisaeidae appear to have “adopted” quill mites from non-passeriform birds. These mites belong to the genera *Gunabopicobia* Skoracki & Hromada, 2013 and *Peristerophila* Kethley, 1970 [47].

The family Corvidae includes some of the most well-known birds in the world. Ravens and crows are particularly renowned for their social behaviour and intelligence. Corvids inhabit nearly every terrestrial environment on Earth, from Arctic tundra and arid deserts to urban streets and tropical rainforests. [48]. Although many species within this family are common, our understanding of the parasitic fauna associated with these birds is quite limited. Of the 130 species in this family, parasitic mites have been recorded on only 12 corvid species (8% of the total), representing just 9 mite species belonging to six genera (including one newly established herein). This underscores the significant gaps in our knowledge regarding the diversity of quill mites that parasitise this group of hosts. A similar situation arises when analysing the quill mite fauna associated with birds of the genus *Corvus*. Unfortunately, our current understanding of the quill mite fauna associated with jackdaws, crows, and ravens also remains far from satisfactory. Presently, only 3 species of syringophilids have been recorded on 4 host species from the genus *Corvus* out of 46 described (constituting just 9%). These are *Syringophiloidus glandarii* (Fritsch, 1958), found on the American Crow, *C. brachyrhynchos* Brehm in the USA, the Rook *C. frugilegus* Linnaeus, and the Eurasian Jackdaw, *C. monedula* Linnaeus in Kazakhstan, *Corvitorotroglus alpha* Skoracki & Bochkov, 2010, found on *C. frugilegus* in Kazakhstan [41,46,49], and *Corvisyringophilus krummi* parasitising the Common Raven, *Corvus corax* in Iceland. Therefore, it is imperative to thoroughly examine the entire genus *Corvus* for the presence of syringophilids. This is crucial for unequivocally describing the host–parasite interactions within the Syringophilidae–*Corvus* system. A future comprehensive analysis of the quill mites associated with birds of the genus *Corvus* will significantly contribute to improving our understanding of syringophilid parasites within the entire Corvidae family.

## 5. Conclusions

This study reveals the complex evolutionary relationships between syringophilid mites and their avian hosts, highlighting their distribution across major avian clades: Palaeognathae, Galloanserae, and Neoaves. Our research introduces the new genus *Corvisyringophilus*, which is closely related to the genera *Kalamotrypetes* and *Colinophilus* found on Galliformes and forms a sister clade to *Blaszakia* and *Charadriphilus*. The distribution of syringophilid mites in early-divergent bird clades supports the hypothesis of multiple host switching followed by co-speciation events.

The limited knowledge of syringophilid fauna associated with hosts of the genus *Corvus* underscores the need for comprehensive examinations of this avian genus to better understand the host–parasite dynamics within the Syringophilidae–*Corvus* system. Additionally, this study highlights the value of museum specimens as a vital resource for taxonomic and ecological analyses, providing insights into the diversity and evolutionary history of syringophilid mites. Museum collections enable comprehensive studies that are often challenging to perform in the field, offering rich opportunities to uncover new species and explore ecological interactions.

## Figures and Tables

**Table 1:** Morphological differences between *Corvisyringophilus* gen. nov. and two closely related genera, *Blaszakia* and *Charadriphilus*.

Character	*Corvisyringophilus*	*Blaszakia*	*Charadriphilus*
Posterior end of stylophore	rounded	rounded	constricted
Hypostomal apex	unornamented	two pairs of protuberances	unornamented
Hypostomal lips	large	small	small
Leg thickness	I thicker than II–IV	I thicker than II–IV	all subequal in thickness
Apodemes I	parallel	slightly divergent	slightly divergent

## Data Availability

Data are available upon request from the corresponding author.

## Supplementary Materials

The following supporting information can be downloaded at https://www.mdpi.com/article/10.3390/ani14192790/s1. Table S1: List of characters used in the phylogenetic analysis. Table S2: Data matrix.
