# Supplementary material for: Corvisyringophilus, a New Genus in the Family Syringophilidae (Acariformes: Prostigmata) and Its Phylogenetic Position among Primitive Genera
Source: Animals (Basel). 2024 Sep 26;14(19):2790. doi: 10.3390/ani14192790 (PMC11475128; doi:10.3390/ani14192790)
Supplement: Supplementary file 1 [file animals-14-02790-s001.zip › animals-3219446-supplementary.pdf]

Suppl S1. List of characters used in the phylogenetic analysis. Character statistics are given for unweighted and unordered datasets.

No.	Character	CI	RI	RC	W
1	Basal part of gnathosoma: not submerged (0); deeply submerged into idiosoma (1)	1	1	1	1
2	Stylophore apodeme: indistinct (0); distinctly developed (1)	1	1	1	1
3	Posterior part of stylophore: constricted (0); rounded (1)	0.2	0.2	0.04	0.1
4	Projection on posterior part of stylophore: absent (0); present (1)	0.25	0.25	0.06	0.17
5	Palpal tibia and tarsus: separated (0); fused (1)	1	1	1	1
6	Claw-like seta of palps: present (0); absent (1)	1	1	1	1
7	Hypostomal apex: unornamented (0); ornamented (1)	0.25	0.75	0.19	0.19
8	Hypostomal protuberances: (0) one pair small and blunt-ended (0); one pair small and sharp-ended (1); two pairs (2); one pair large (3)	0.6	0.33	0.2	0.2
9	Size of hypostomal lips: small (0); large (1)	0.5	0.5	0.25	0.25
10	Shape of peritremes: M-shaped (0); U-shaped (1)	1	1	1	1
11	Lateral branch of peritreme: normal i.e., 6-10 chambers (0); reduced i.e, 1-5 chambers (1); supernumerary i.e, 11-20 chambers (2)	0.2	0.33	0.06	0.13
12	Distal tip of chelicerae: edentate (0); dentate (1)	0.33	0.87	0.29	0.29
13	Position of setae *vi* and *ve*: at same level (0); vi anterior to ve (1)	0.5	0.5	0.25	0.25
14	Position of setae *c1* and *se*: at same level (0); not at same level (1)	0.25	0.4	0.1	0.1
15	Position of setae *c1* and *se*: *se* slightly anterior to *c1* (0); *se* distinctly anterior to *c1* (1); *se* posterior to *c1* (2)	0.67	0.9	0.6	0.4
16	Position of setae *se* and *c2*: *se* posterior to *c2* (0); *se* and *c2* at same level (1)	0.33	0.33	0.11	0.11
17	Position of setae *se*: on propodonotal shield (0); out of shield (1)	0.4	0.7	0.28	0.28
18	Position of setae *c2*: out of shield (0); on shield (1)	0.50	0.67	0.33	0.33
19	Propodonotal shield: strongly (0); weakly sclerotized (1)	0.5	0	0	0
20	Hysteronotal shield: present (0); absent (1)	0.17	0.17	0.03	0.03
21	Hysteronotal shield: entire (0); divided (1)	1	1	1	1
22	Pygidial shield: present (0); absent (1)	0.5	0	0	0
23	Hysteronotal shield: reach level of setae *d2* (0); not reach *d2* (1)	0.17	0.5	0.08	0.12
24	Hysteronotal and pygidial shields: fused (0); not fused (1)	0.33	0.33	0.11	0.11
25	Position of setae *f1* and *f2*: *f1* close to *f2* (0); *f2* far from *f1* (1)	0.5	0.86	0.43	0.43
26	Position of setae *ag1* and *ag2*: *ag2* postero lateral to *ag1* (0); *ag2* posterior to *ag2* (1); *ag1* and *ag2* at same level (2)	0.33	0.67	0.22	0.22
27	Neotrichious aggenital setae: absent (0); present (1)	0.5	0.67	0.33	0.33
28	Setae *ps3*: present (0); absent (1)	1	1	1	1
29	Setae *e1*: present (0); absent (1)	1	1	1	1
30	Position of setae *3a* and *3b*: at same level (0); *3a* anterior to *3b* (1)	1	1	1	1
31	Setae *4a*: present (0); absent (1)	1	1	1	1
32	Ornamentation of idiosomal setae: present (0); absent (1)	0.25	0.5	0.13	0.22
33	Seta *vs* on tarsus III: present (0); absent (1)	0.5	0.5	0.25	0.25
34	Seta *vs* on tarsus IV: present (0); absent (1)	1	1	1	1
35	Seta *dG* on genu IV: present (0); absent (1)	1	1	1	1
36	Seta *vF* on femur III: present (0); absent (1)	1	1	1	1
37	Seta *v* on trochanter III: present (0); absent (1)	1	1	1	1
38	Proral setae p' and p": rod-like (0); fan-like (1)	1	1	1	1
39	Number of tines in proral setae: reduced i.e, 4-10 tines (0); normal i.e, 11-20 tines (1); supernumerary 21-35 tines (2)	0.33	0.69	0.23	0.23
40	Legs I and II: subequal in thickness (0); I thicker than II (1)	0.5	0.75	0.38	0.38
41	Legs I and II: I longer than II (0); I and II subequal in length (1)	1	1	1	1
42	Coxal fields I-II and III-IV: grouped together (0); widely separated (1)	1	1	1	1
43	Apodemes I and II: similar in shape (0); different in shape (1)	0.33	0.6	0.2	0.2
44	Apodemes I: divergent (0); parallel (1)	0.5	0.75	0.38	0.38
45	Divergence of apodemes I: strongly divergent (0); slightly divergent (1)	0.5	0.91	0.45	0.45
46	Apodemes I and II: not fused (0); fused (1)	0.33	0.67	0.22	0.22
47	Apodemes I and II: fused in posterior part of apodemes II (0); fused in middle part of apodemes II (1)	1	1	1	1
48	Apodemes III and IV: present (0); absent (1)	0.5	0.83	0.42	0.42
49	Claw basal angle: absent (0); present (1)	1	1	1	1
50	Body size: large i.e., more than 1000 (0); medium i.e., 700-990 (1); small i.e., less than 690 (2)	0.25	0.63	0.16	0.13

Suppl. S2. Data matrix.

	**1** 2 3 4 5
	12345678901234567890123456789012345678901234567890
Cheyletus eruditus (**outgr**.)	00--000-000000-0000000000000000000000000-0000000-000
Cheletopsis norneri (**outgr**.)	00--000-000001101001-1--00000000000000-0000000-000
Aulobia dendroicae	1100110-102010-00000001000011111111111001111-0-101
Aulobia cardueli	1100110-102010-00000001000011111111111001111-0-101
Aulobia sylviae	11101110002010-00000001100011111111111001111-0-101
Corvisyringophilus krummi	1110110-1000112000000010110111111111111011111-0-102
Blaszakia rossae	111011120000112000000000110111101111110111010-102
Bubophilus aegolius	1100110-001110-00000001001011111111111110111010-101
Charadriphilus ludmilae	1100110-00201120000000001101111011111100111010-102
Charadriphilus ralli	1100110-00001120010000001101111011111100111010-102
Colinophilus wilsoni	1100110-002111200000000000101111111111111111010-100
Corvitorotroglus alpha	1100111100011110100000100-111111111111101110011100
Creagonycha totani	1100110-000111111011-0--0101101111111110110000-100
Crotophagisyringophilus io	11001111002111102001-0--01011111111111101110111100
Ixobrychiphilus wallacei	1100110-0010111000100011010111111111111001111-0-102
Kalamotrypetes cracidus	1111110-00001120000000000001111111111101111010-102
Kalamotrypetes colinastes	1111110-00200120000000000001111111111101111010-101
Megasyringophilus eos	11001112001111101001-1--1001101111111110111000-010
Megasyringophilus trichoglossus	1100111?000111102000001010011011111111110111000-010
Megasyringophilus aquilus	11001111000111102000001010011011111111120111000-010
Pteroclidisyringophilus otididus	1100110-0010110000000001000011111111111110111010-102
Pteroclidisyringophilus re	1100110-0010112001000000000111111111111110111010-102
Selenonycha charadriiformicus	1110111-000111111100000101101101111111110110000-000
Syringophilopsis turdi	11001112002111101001-0--020111111111111001110011100
Syringophilopsis muscicapicus	1100111200211110100010110101111111111111001110011100
Syringophilus bipectinatus	1100110-0100110001000000000111111111111101110110101
Syringophilus numidae	1100110-0100110001000000000111111111111201110110100
Tinamiphilopsis ariconte	1101110-000111000000000000101111011111110111000-102
Tinamiphilopsis elegans	1101110-00011110000000100101111011111110111000-101
Torotrogla meulae	1100111300011111100010110-11111111111110110000-101
Torotrogla lusciniae	11001113000111111001-0--0-11111111111110110000-001
Trypetoptila casmerodia	11011112000110-00001-0--0-111111011111201110011100
